# Molecular Mechanisms, Epidemiology, and Clinical Importance of β-Lactam Resistance in *Enterobacteriaceae*

**DOI:** 10.3390/ijms21145090

**Published:** 2020-07-18

**Authors:** Giulia De Angelis, Paola Del Giacomo, Brunella Posteraro, Maurizio Sanguinetti, Mario Tumbarello

**Affiliations:** 1 Dipartimento di Scienze Biotecnologiche di Base, Cliniche Intensivologiche e Perioperatorie, Università Cattolica del Sacro Cuore, 00168 Rome, Italy; giulia.deangelis@unicatt.it (G.D.A.); brunella.posteraro@unicatt.it (B.P.); maurizio.sanguinetti@unicatt.it (M.S.); 2 Dipartimento di Scienze di Laboratorio e Infettivologiche, Fondazione Policlinico Universitario A. Gemelli IRCCS, 00168 Rome, Italy; paola.delgiacomo@gmail.com; 3 Dipartimento di Scienze Gastroenterologiche, Endocrino-Metaboliche e Nefro-Urologiche, Fondazione Policlinico Universitario A. Gemelli IRCCS, 00168 Rome, Italy; 4 Dipartimento di Sicurezza e Bioetica, Università Cattolica del Sacro Cuore, 00168 Rome, Italy

**Keywords:** *Enterobacteriaceae*, β-lactamase, β-lactam drugs, molecular resistance

## Abstract

Despite being members of gut microbiota, *Enterobacteriaceae* are associated with many severe infections such as bloodstream infections. The β-lactam drugs have been the cornerstone of antibiotic therapy for such infections. However, the overuse of these antibiotics has contributed to select β-lactam-resistant *Enterobacteriaceae* isolates, so that β-lactam resistance is nowadays a major concern worldwide. The production of enzymes that inactivate β-lactams, mainly extended-spectrum β-lactamases and carbapenemases, can confer multidrug resistance patterns that seriously compromise therapeutic options. Further, β-lactam resistance may result in increases in the drug toxicity, mortality, and healthcare costs associated with *Enterobacteriaceae* infections. Here, we summarize the updated evidence about the molecular mechanisms and epidemiology of β-lactamase-mediated β-lactam resistance in *Enterobacteriaceae*, and their potential impact on clinical outcomes of β-lactam-resistant *Enterobacteriaceae* infections.

## 1. Introduction

As a class of antimicrobial drugs that have revolutionized our ability to treat bacterial infections, since the introduction of penicillin into the clinic in the 1940s [1], β-lactams remain the cornerstones of today’s antibacterial armamentarium [2]. These drugs interfere with bacterial cell wall biosynthesis because they covalently inhibit transpeptidases, namely penicillin-binding proteins (PBPs), through the acylation of an active site serine in these essential enzymes for the growth of replicating bacteria [3]. As discussed below, most β-lactamases contain an active site serine that can be acylated by β-lactam molecules, thus accounting for structural and mechanistic commonalities between the two acyl-enzyme types (i.e., PBPs and β-lactamases) [4].

At present, β-lactams are the most widely prescribed antibiotics [5] and comprise four main groups for therapeutic use. Three groups share a bicyclic structure (i.e., penicillins, cephalosporins, and carbapenems) and the fourth group has a monocyclic structure (i.e., monobactams). In one case, the four-membered 2-azetidinone ring (i.e., the β-lactam ring) fuses either to a thiazolidine ring (penicillins) or to a six-membered dihydrothiazine (cephalosporins), or completes a five-membered pyrroline (carbapenems). Extensive programs of modification of the natural products identified in each group led to create arrays of semi-synthetic derivatives [2]. In parallel, substantial developments would allow for improvements in the potency, pharmacokinetics, safety, and spectrum of activity of β-lactam-containing antibacterial agents, and, importantly, to address the specific resistance mechanisms that were arising in the bacterial species targeted by these agents [6].

**Figure 1:**
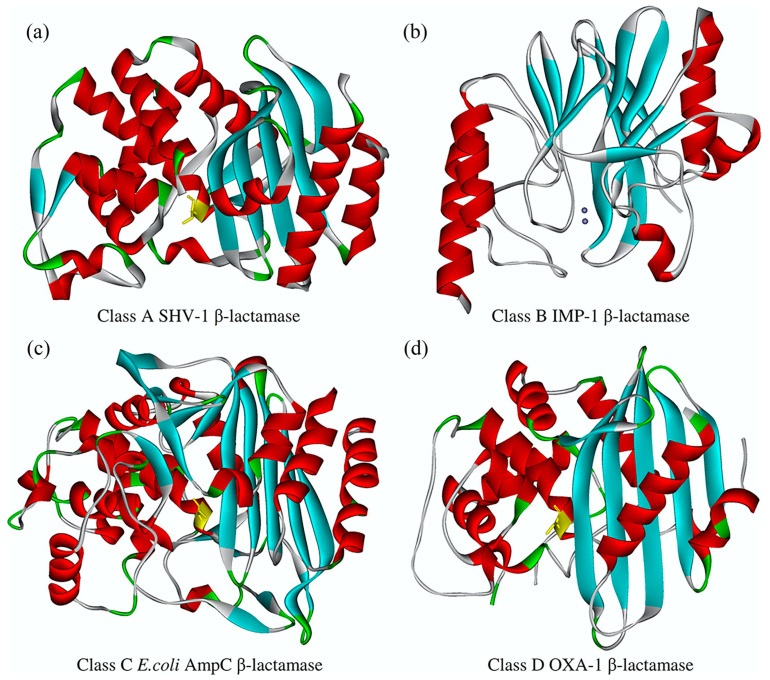
Overall structure of representative β-lactamases from A, B, C, and D classes (adapted from reference 92 with permission). (**a**) Class A SHV-1 (sulfhydryl reagent variable-1); (**b**) class B IMP-1 (imipenem-resistant Pseudomonas-1); (**c**) class C (AmpC (ampC β-lactamase); (**d**) class D OXA-1 (oxacillinase-1). Active sites of the serine-β-lactamases are colored yellow, and the metallo-β-lactamase zinc ions are shown as gray spheres.

In Gram-negative bacteria—including *Enterobacteriaceae*, a family encompassing many clinically relevant species—the production of β-lactamases that hydrolyze the β-lactam ring, thereby inactivating the drug, is the predominant cause of resistance to β-lactams [7]. These enzymes are grouped into four classes (A, B, C, and D) based on amino acid sequences [8]. Except for class B metalloenzymes, commonly referred to as metallo-β-lactamases (MBLs), class A, C, or D β-lactamases belong to the family of serine-reactive hydrolases [9]. Consistently, the hydrolytic process is via either a covalent acyl-enzyme intermediate formed between the β-lactam molecule and the active site serine, or a reaction facilitated by the presence of one or two zinc ions in the MBL active site [10]. Figure 1 depicts some representative class A, B, C, or D enzymes with indicated key catalytic residues of serine-β-lactamases and metallo-β-lactamase zinc ions, respectively. In the first case, a reactive water molecule hydrolyzes the acyl-enzyme intermediate and, in the second case, a hydroxide ion directly attacks the carbonyl carbon of the amide [11]. Following the release of the product of hydrolysis, the regeneration of the active site allows for the next turnover.

With the therapeutic use of safe and effective β-lactam antibiotics, the prevalence and variety of β-lactamases have multiplied dramatically during the last decades, and the evolution of β-lactamases found in clinical isolates has become the focus of some notable reviews recently published [7,10]. Although there are almost 4900 β-lactamases, as unique enzymes, identified that are potentially able to hydrolyze β-lactam antibiotics [12], the number of β-lactamases continues to increase considerably, presumptively owing to the easy access to inexpensive and rapid gene sequencing [10]. Of these enzymes, extended-spectrum β-lactamases (ESBLs) and carbapenemases are attracting the largest amount of current clinical interest [13], particularly because infections caused by the ESBL-producing *Enterobacteriaceae* (ESBL-E) and carbapenemase-producing *Enterobacteriaceae* (CPE) are associated with an increased mortality, time to effective therapy, length of stay (LOS), and healthcare costs [13,14].

While the 20th century “antibiotic golden-age” is ending [15], the rates of antimicrobial resistance (AMR) are rising globally, and patient deaths resulting from AMR are projected to reach 10 million annually by 2050 [16]. Notably, the WHO published in 2017 a global priority pathogens list that included species of the *Enterobacteriaceae* family as the most significantly resistant pathogens. In particular, *Enterobacteriaceae* resistant to third-generation cephalosporins (ESBL-E, among others) and *Enterobacteriaceae* resistant to carbapenems (CRE) were listed [17]. Thus, pathogens of this nature, many of which harbor acquired multidrug resistance plasmids, can transmit antimicrobial resistance genes on an intra- and inter-species level, thereby complicating infection control actions and exacerbating the need for new therapeutic treatments [18,19].

The aim of this review is to focus on the molecular mechanism and epidemiology of the β-lactamase-mediated resistance to β-lactam antibiotics in *Enterobacteriaceae* as well as on the clinical consequences associated with such antimicrobial resistance.

## 2. β-Lactam Molecular Resistance in *Enterobacteriaceae*: β-Lactamases

**Figure 2:**
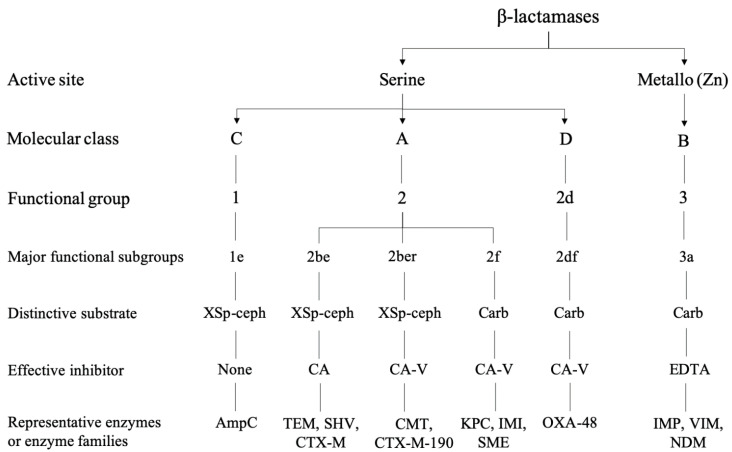
Molecular and functional relationships among β-lactamases conferring resistance to selected cephalosporins or carbapenems (adapted from reference 10 with permission). XSp-ceph, expanded-spectrum cephalosporins; Carb, carbapenems; CA, clavulanic acid; CA-V, variable response to clavulanic acid; EDTA, ethylene diamine tetra-acetic acid.

Although there are three major molecular mechanisms, such as enzyme production, efflux pump overexpression, and porin modification, by which *Enterobacteriaceae* (and other Gram-negative bacteria) develop resistance to β-lactam-containing antibacterial agents [20], the enzymatic inactivation is predominant [21] and, alone or combined with the others, is associated with the multidrug-resistant phenotypes observed in clinical isolates [22]. The combination of both molecular and functional characteristics, based on the groupings respectively developed by Ambler [8] and Bush and Jacoby [23], enabled a more comprehensive classification scheme for the four major β-lactamase classes [7]. As shown in Figure 2, AmpC (ampC β-lactamase) cephalosporinases, ESBLs, inhibitor-resistant enzymes, and carbapenemases can be classified into functional subgroups (1 and 1e into class C; 2a, 2b, 2be, 2br, and 2f into class A; 2de and 2df into class D; 3a and 3b into class B), according to different substrate hydrolysis and inhibitor profiles. Class D β-lactamases are enzymes that cause carbapenem resistance—resulting from the combined action of an OXA (oxacillinase) type carbapenemase and a non-enzyme resistance mechanism—especially in non-*Enterobacteriaceae* organisms such as *Acinetobacter baumannii* and *Pseudomonas aeruginosa* [24].

Notably, ESBLs include not only the TEM (Temoneira β-lactamase) and SHV (sulfhydryl reagent variable β-lactamase) families of ESBLs (subgroup 2be), but also cephalosporinases with expanded substrate hydrolysis profiles (subgroup 1e), the CTX-M (cefotaxim-hydrolizing β-lactamase) family of ESBLs (subgroup 2be), and the cephalosporin-hydrolyzing OXA enzymes (subgroup 2be). Likewise, carbapenemases include both serine carbapenemases (subgroups 2f and 2df) and MBLs (subgroups 3a and 3b). As different β-lactam-containing antibacterial agents were introduced for the treatment of bacterial infections [6], the well-known hydrolytic spectrum of ESBLs was extended to include expanded-spectrum cephalosporins among other agents (i.e., penicillins, cephalosporins, monobactams). It is noteworthy that, like ESBLs, most carbapenemases hydrolyze many expanded-spectrum cephalosporins. Despite this, it is clinically important to keep a clear distinction between carbapenemases and ESBLs, as it was suggested in [23].

### 2.1. Extended-Spectrum β-Lactamases

Among class A ESBLs, SHV- and TEM-type enzymes comprise almost 240 variants each [11,25], which are mainly plasmid-encoded. These variants originated from parental enzymes (i.e., SHV-1, TEM-1, or TEM-2) following amino acid substitutions due to point mutations in the parental genes. This occurred a few years after the introduction of new β-lactams substituted with an oxyimino side chain (e.g., aztreonam and the third-generation or expanded-spectrum cephalosporins) into clinical practice [26,27]. As oxyimino-cephalosporins are good PBP inhibitors but poor substrates for broad-spectrum β-lactamases, it is not unexpected that variants of class A enzymes gained the ability to hydrolyze oxyimino-cephalosporins, such as cefotaxime and ceftazidime [6]. As a new subgroup of class A ESBLs that emerged in 2000 [28], CTX-M-type enzymes are now the most prevalent ESBLs in *Enterobacteriaceae* worldwide [29], with more than 230 variants subdivided in five major clusters (CTX-M-1, CTX-M-2, CTX-M-8, CTX-M-9, and CTX-M-25) [30]. Unlike the TEMs and SHVs that originated from plasmid-encoded penicillinases, the CTX-M enzymes are related to chromosomal β-lactamases from different species of *Kluyvera* [31]. This suggests that insertion sequences and, to a lesser extent, bacteriophages mobilize *bla*_klu_ genes into conjugative plasmids [32]. Other plasmid-encoded class A ESBLs found in *Enterobacteriaceae* are SFO (*Serratia fonticola* β-lactamase), TLE (TEM-like enzyme), PER (Pseudomonas extended resistant β-lactamase), BES (Brazil extended-spectrum β-lactamase), and GES (Guiana extended-spectrum β-lactamase) enzymes [33,34,35,36].

As in the other class A enzymes that also include β-lactamases with carbapenemase activity (see below), the ESBLs’ active sites are located between two β and α/β domains, accommodating a Ω loop structure [11]. In particular, Glu166 and Asn170 amino acids play an important role in catalyzing β-lactam substrates, with Glu166 activating the catalytic water for the deacylation step and with Asn170 helping Glu166 to reach the proper position in order to activate the water. Other essential residues for substrate binding and catalysis (e.g., Ser70, Lys73, Ser130, and Lys234, etc.) are strictly conserved in all class A β-lactamases [11]. As both acylation and deacylation require the activation of the nucleophilic serine (Ser70) and hydrolytic water, respectively, Glu166 is seemingly involved as the activating base in both acylation and deacylation [37]. Nevertheless, the leading role of Glu166 in the acylation step is debatable, and data suggest the key function of Lys73 residue. Studying the dynamic interaction between TEM-1 and penicillanic acid, Meroueh et al. noticed that Lys73 acts as a general base for serine activation concertedly with Glu166 [38]. Seminal studies suggest that class A β-lactamases are “perfect enzymes”, and this may be partly due to their ability to distinguish between substrates and products [39]. The substrate recognition influences the efficiency of acylation, whereas the product release influences the absence of inhibition by the product, which is a key feature of β-lactamases [40].

We now understand that TEM and SHV variants differ by no more than two or three amino acids in the coding region, and this extended the hydrolytic spectrum from penicillins and narrow-spectrum cephalosporins to expanded-spectrum cephalosporins. As recently reviewed by Palzkill et al. [11] and Liakopoulos et al. [25], the amino acid substitutions in TEM-1/TEM-2 and SHV-1 enzymes that underpin the ESBL phenotypes in clinical isolates are located in key elements of the binding site, namely the above mentioned Ω loop (amino acids 160–181) and the β-strand β3 (amino acids 229–240). Although G238S and R164S substitutions are core substitutions of the TEM/SHV ESBLs, they frequently combined with additional point mutations (e.g., E104K, M182T, A237T, and E240K). Both G238S and R164S are associated with the conformational changes of the Ω loop or the β-strand β3 that result in expanding the binding site and potentially facilitating the accommodation of oxyimino-cephalosporins in the enzyme’s active site [41,42]. This takes place by the movement of the β-strand β3 or the Ω loop due to the G238S substitution, and by the creation of a cavity in the middle of the Ω loop due to the R164S mutation. Interestingly, the M182T substitution–a well-known suppressor of folding and stability defects [43]–increases the catalysis of oxyimino-cephalosporins, despite involving the amino acid 182 which is not located in the vicinity of the active site, probably compensating for the above mentioned ESBL substitutions [44].

The CTX-M enzymes are identical to the SHV or TEM enzymes for only 40% or less of their amino acid sequences, which contrasts with a 70% or higher identity within the subgroup [43,45]. Accordingly, CTX-M enzymes differ from most β-lactamases by an increased hydrolytic activity against cefotaxime [46], suggesting that the ability to hydrolyze expanded-spectrum cephalosporins is a subgroup’s intrinsic enzymatic property rather than the result of point mutations [45]. Thus, studies showed that the Ser237 and Arg276 amino acids both contribute to the CTX-M specificity for cefotaxime hydrolysis [40,47], supporting the finding that this amino acid combination is absent in other class A β-lactamases [48]. In particular, Delmas et al. [40] showed that the binding of cefotaxime induces subtle conformational changes in the CTX-M active site, and these changes cause the rupture of the hydrogen bond between Asn170 and Asp240, which connects the Ω loop to the β-strand β3. Importantly, Adamski et al. [47] used the crystal structures of amino acid mutants alone and in complex with cefotaxime to show that Ser237 and Arg276, in the wild-type CTX-M enzyme, cooperate to shape the small active site in the region of Asn170 and Asp240 in order to accommodate cefotaxime. Compared to cefotaxime, the related oxyimino-cephalosporin ceftazidime is poorly hydrolyzed [45]. Nonetheless, studies showed that the D240G and P167S amino acid substitutions increase the ability of wild-type CTX-M to hydrolyze ceftazidime, with both the mutations altering the position of the aminothiazole ring into the active site and, thence, allowing a better contact with ceftazidime [49,50]. Conversely, substitutions involving amino acids not located in the vicinity of the active site (i.e., A77V and N106S) increase the hydrolysis of oxyimino-cephalosporins only when they are combined with the P167S or D240G substitutions [51,52]. It is possible that, similar to TEM/SHV M182T, these amino acid substitutions enhance the enzyme thermostability as well as in vivo expression levels.

As recently reviewed by Bonomo [22], class A enzymes are usually susceptible to inactivation by clinically available β-lactamase inhibitors, such as clavulanate, sulbactam, tazobactam, and avibactam. To explain the mechanism underlying this inactivation, extensive work on TEM/SHV ESBLs suggests that either amino acid substitutions leading to inhibitor-resistant variants (or variants with higher hydrolytic capacities) or an enzyme hyperproduction may be involved [53,54]. As the substitutions occur in parental TEM-1/TEM-2 enzymes with concurrent ESBL-defining substitutions, the resulting enzymes are termed complex mutant TEM (CMT) β-lactamases (e.g., TEM-50, TEM-151, TEM-152, and TEM-158) [55]. A very recent study by Rodríguez-Villodres [56] confirmed the latter hypothesis, showing that exposing *bla*_TEM_-carrying *Escherichia coli* clinical isolates to piperacillin/tazobactam induced an increase in the copy numbers and transcription levels of the *bla*_TEM_ gene. Interestingly, genome sequencing revealed the presence of *bla*_TEM_ gene duplications in two *bla*_TEM_-carrying representative isolates (8 and 60 copies, respectively).

The CTX-M-190 is the first inhibitor-resistant CTX-M variant detected in clinical *E. coli* isolates that, together with CTX-M-199, retained hydrolytic activity on expanded-spectrum cephalosporins [57,58]. These variants arose due to the Ser-to-Thr amino acid substitution at positions 130 of CTX-M-55 and CTX-M-64, respectively. Very recently, Cheng et al. [59] provided structural insights into the inhibitor resistance mechanism in CTX-M-199. Results from mass spectrometry and crystallography analyses did not reveal any structural modification of the active site in CTX-M-64 (S130T) compared to the CTX-M-64 enzyme. However, the binding of the sulbactam to the active site rendered the formation of the inhibitor–enzyme complex ineffective.

### 2.2. Carbapenemases

Class A carbapenemases include the KPC (*Klebsiella pneumoniae* carbapenemase)-, SME (*Serratia marcescens* enzyme)-, Nmc-A (non-metallo-carbapenemase-A)-, IMI (IMIpenemase)-, and GES-type enzymes [21]. They may be chromosomally encoded (SME and Nmc-A), plasmid-encoded (KPC and GES), or both (IMI) [60]. As mentioned above, all class A β-lactamases with carbapenemase activity, such as KPC, avoid inhibitory interactions with carbapenems [6]. According to recent studies, 54 subtypes of KPC [12], 43 subtypes of GES [12], 5 subtypes of SME [12,61], and 19 subtypes of IMI [12,62] exist globally. Recently, CTX-M enzymes gained carbapenemase activity, and Poirel et al. [63] who identified a CTX-M-15 derivative (CTX-M-33) in a clinical *K. pneumoniae* isolate documented it. Compared to CTX-M-15, the CTX-M-33 enzyme harbored an Asn to Ser substitution at position 106. The structural data on such enzymes show that the possession of a disulfide bridge between amino acids 69 and 238 enables class A carbapenemases to be distinguished from other class A β-lactamases [64]. Although this disulfide is structurally important [65], it appears to be not essentially required for carbapenemase activity [6]. Indeed, the disulfide disruption by mutation destabilizes the Nmc-A enzyme [66], whereas a C69G mutant of GES-5, though destabilized, remains catalytically competent [67]. By contrast, it has become clear that the specific spatial requirements on the class A β-lactamase active site–likely coupled with the Cys69–Cys238 disulfide bridge–are necessary to orient bound carbapenems for hydrolysis, and these requirements may be more stringent than for other β-lactam substrates [6]. In this context, Tooke et al. [6] suggested a possible association between active site expansion and reduced carbapenemase activity, which would be consistent with the fact that KPC subtypes with increased activity against the (notably bulky) ceftazidime showed reduced activity against carbapenems [68]. Furthermore, GES enzymes lacking Ser170, such as GES-1, are unable to hydrolyze carbapenems, whereas the hydrogen bonds to Ser170 need to persist to maintain Glu166 in a position and orientation necessary for deacylation [6].

The class B, zinc-dependent MBLs belong to a large superfamily of metallohydrolases that act upon a range of microbial substrates spanning from small molecules to nucleic acids [6]. In MBLs, a metal center is located at the interface of two β-sheets that form a protein core, and both the center’s architecture and the amino acids composing it define three distinct MBL subclasses, namely B1, B2, and B3. As recently reviewed by Tooke et al. [6], B1 enzymes and B3 enzymes possess a binuclear zinc center, which comprises Zn1 (tri-His) and Zn2 (Cys–His–Asp (B1) and His–His–Asp (B3)) metal sites. The active site mobile loops, including a hydrophobic L3 loop and a hydrophilic L10 loop–both of which are involved in substrate binding–flank the two bivalent zinc ions [69]. Of the two water molecules that complete the zinc coordination, one (the so-called “bridging” water) connects the two zinc ions and the other (the so-called “atypical” water) is bound to Zn2. As β-lactamases, the binuclear enzymes have an exceptionally broad spectrum of activity that encompasses penicillins, cephalosporins, and carbapenems [70]. Monobactams are the unique exceptions. Unlike class A carbapenemases, MBLs are not inhibited by β-lactamase inhibitors [71]. The most clinically relevant MBLs–acquired MBLs, encoded by DNA on mobile elements—are the B1 subclass enzymes NDM (New Delhi MBL), VIM (Verona IMipenemase), and IMP (imipenem-resistant *Pseudomonas*) [69,72]. According to recent studies, 29 NDM variants [12,73], 69 VIM variants [10,12,73], and 85 IMP variants [10,12,74] exist globally. Only a minority of VIM or IMP variants occur in *Enterobacteriaceae* [69].

Despite extensive investigation, the catalytic mechanism of MBLs remains incompletely understood, partly because of the sequence/structural diversity (within even the same MBL subclass) that complicates the identification of mechanistic commonalities. Recently, the crystal structures of NDM-1, in a complex with hydrolyzed carbapenems (imipenem and meropenem), revealed that the NDM-1-catalyzed carbapenem hydrolysis proceeds through a peculiar mechanism, probably involving the protonation of hydrolytic intermediates by a bulky water molecule incoming from the β-face [75]. Consistent with these findings, a deep sequencing study of NDM-1 identified a number of positions at which mutation affected carbapenem hydrolysis much more strongly than that of other β-lactam substrates [76]. Furthermore, Palacios et al. [77] investigated the role of the loop L3–a mobile flap shown to adopt different conformations upon substrate or inhibitor binding. Interestingly, the study showed that the loop replacement in the scaffold of NDM-1 did not shape the enzyme’s substrate profiles but did affect the catalytic mechanism, interfering with the protonation rate of anionic reaction intermediates. Unlike other MBLs, the VIM enzymes display variations at positions 224 and 228, which may be key positions for the interaction with β-lactam substrates [78,79,80]. Using the crystal structures of the VIM-1 enzyme and its complexes–one with a thioenolate inhibitor and the other with the hydrolyzed carbapenem—Salimraj et al. [81] showed that both complexes share a water-mediated hydrogen bond between the carboxylate group of the substrate/inhibitor and the backbone carbonyl of the active site of the Zn2 ligand Cys221. The authors suggested that Cys221 might replicate the role of the conserved Lys224 in analogous complexes with other MBLs [81].

Initially identified as enzymes with activity restricted to penicillins, class D β-lactamases were categorized as “oxacillinases” because of their ability to hydrolyze oxacillin at a rate of at least 50% that of benzylpenicillin [21]. The OXA class now encompasses different enzymes (more than 250 enzymes) active against penicillins, cephalosporins, expanded-spectrum cephalosporins (OXA-type ESBLs), and carbapenems (OXA-type carbapenemases), with widely differing sensitivities to inhibitors [82]. Of the five recognized groups of OXA carbapenemases, four (OXA-23, OXA-24/40, OXA-51, and OXA-58) are primarily associated with resistance in *A. baumannii*. In contrast, OXA-48-type β-lactamases are found on plasmids in *Enterobacteriaceae* [83]. OXA-48 enzymes share more than 90% of their amino acid identity with the chromosomally encoded oxacillinase genes from the aquatic *Shewanella* species, suggesting that *Shewanella* enzymes are likely the ancestors of OXA-48 enzymes [84]. OXA β-lactamases use the carboxylation of the conserved active site lysine (the equivalent of Lys73 in class A β-lactamases) as the key determinant of activity. Indeed, the carboxylated lysine activates the nucleophilic serine used for β-lactam hydrolysis in a near equivalent position to that of Glu166 in class A β-lactamases, and forms the base for both the acylation and deacylation reaction steps [85]. Subtle structural changes in the active site enabled carbapenem-hydrolyzing class D β-lactamases (CHDLs) to evolve the ability to hydrolyze the imipenem. The substrate kinetics and mechanistic characterization of a prominent CHDL, the OXA-58 enzyme, from *A. baumanni* revealed that the removal of steric hindrances from the path of the deacylating water molecule might contribute to a tighter binding of imipenem to the enzyme’s active site [86]. Multiple studies aiming to elucidate the interactions between carbapenems and OXA carbapenemases have indicated that a hydrophobic bridge over the active site is an important structural element for the activity against carbapenems [87,88]. In contrast, OXA-48 enzymes–that are only distantly related to the *A.* *baumanni* CHDL enzymes–lack an active site hydrophobic bridge, and this observation is consistent with the retention of the activity against oxacillin, which is a poor substrate for other OXA carbapenemases [89]. Furthermore, Smith et al. [90,91] showed that *A. baumanni* CHDLs are able to expel the water from the active site on acylation, resulting in the recruitment of a deacylating water molecule from the milieu. Consequently, the carbapenem turnover may be also dependent on the access of water to the active site, perhaps through conformational changes in the acyl-enzyme [88]. In particular, the water molecule enters the active site through a channel formed by the displacement of conserved hydrophobic surface amino acids. A more recent study conducted by Smith et al. [91] showed that the channel was pre-existing in OXA-48 despite only observing a minor movement of the above-mentioned amino acids that allowed for the enlargement of the channel.

### 2.3. Extended-Spectrum AmpC Cephalosporinases

Although class C AmpC β-lactamases are usually encoded by chromosomal *ampC* genes (e.g., CMY-2, P99, ACT-1, and DHA-1) in the *Enterobacter* and *Citrobacter* species, plasmid-borne AmpC enzymes are more prevalent in the *Klebsiella* and *Salmonella* species among *Enterobacteriaceae* family members [92]. The production of AmpC enzymes is low (i.e., “repressed”) or inducible (i.e., “derepressed”, following an induction with, for example, cefoxitin), thus conferring resistance to aminopenicillins and early-generation cephalosporins (cephalothin, cefuroxime, and cefoxitin). However, spontaneous mutations in the AmpC regulatory genes, inducing the constitutive AmpC overproduction, result in organisms displaying a resistance to expanded-spectrum cephalosporins, such as the oxyimino-cephalosporins cefotaxime, ceftriaxone, and ceftazidime. AmpC enzymes poorly hydrolyze cefepime and are inhibited by cloxacillin, oxacillin, and aztreonam [92]. In *E. coli*, because of a weak promotor and the presence of a transcriptional attenuator, the expression of the chromosomally encoded AmpC is undetectable but behaves constitutively in the presence of plasmid-mediated *ampC* genes (e.g., *bla*_CMY_, *bla*_FOX_, *bla*_DHA_, *bla*_ACC_, *bla*_ACT_, *bla*_MIR_, *bla*_MOX_, etc.) [93].

Despite being structurally related to narrow-spectrum cephalosporinases, the extended-spectrum AmpC (ESAC) β-lactamases differ by amino acid insertions, deletions, or substitutions [93]. Like ESBLs, ESAC β-lactamases efficiently hydrolyze penicillins, cephamycins, and third-generation cephalosporins but are inactive against carbapenems. Unlike ESBLs, ESAC β-lactamases are not inhibited by conventional β-lactamase inhibitors. Since the first description in 1989 [94], plasmid-mediated AmpC β-lactamases have been classified into several families (CMY (cephamycin-hydrolyzing β-lactamase), MIR (Miriam Hospital β-lactamase), MOX (moxalactam-hydrolyzing β-lactamase), LAT (latamoxef-hydrolyzing β-lactamase), FOX (cefoxitin-hydrolyzing β-lactamase), DHA (Dhahran Hospital in Saudi Arabia β-lactamase), ACT (AmpCtype β-lactamase), ACC (Ambler C class β-lactamase), CFE (*Citrobacter freundii* β-lactamase)), which display small amino acidic differences, especially in *K. pneumoniae* isolates [95]. Different mutations of both chromosomally (i.e., GC1 of *E. cloacae*) and plasmid-encoded (i.e., CMY-10) AmpC β-lactamases enhance the catalytic efficiency toward oxyimino-β-lactam substrates (subgroup 1e, Figure 2).

The molecular structure of AmpC β-lactamases includes a small helical domain with three α-helices and loops, and a mixed α/β domain. The active site accommodates the R1 region, which is surrounded by the Ω-loop and interacts with the C7 side chain of β-lactams, and the R2 region, which is surrounded by the R2 loop with the helix H-10 and H-11 therein. Modifications of the wild-type AmpC gene into key regions close to Ω-loop, namely the H-10 and H-11 helices, are associated with the ESAC β-lactamase activity [93]. One study described the in vivo evolution of CMY-2 (the most common plasmid-mediated AmpC β-lactamase) to an extended-spectrum AmpC β-lactamase (CMY-33) in clonally identical *E. coli* isolates, which were obtained from a patient previously treated with cefepime. The CMY-33 enzyme was shown to be different from the CMY-2 due to a Leu293–Ala294 deletion in the H-10 helix. This caused a modification in the size and, perhaps, the flexibility of the active site, following an augmentation of the distance from Ser64 to the H-10 helix [96]. Recently, these findings were supported by an in vitro study with an *E. coli* strain expressing a CMY-2-encoding plasmid in subinhibitory concentrations of cefepime [97]. The CMY-2 enzyme evolved to CMY-69 (due to the Ala294Pro substitution in the H-10 helix) after 24 passages, whereas the cefepime minimum inhibitory concentration (MIC) value gradually increased to reach a high resistance level (MIC, >256 mg/L) after 30 passages. In parallel, the mutant strains derived from the wild-type CMY-2 strain exhibited a progressive increase in the *ampC* gene transcription, which correlated with an increase in the plasmid copy number and the higher MIC values observed in vitro [97].

## 3. β-Lactamase-Mediated Resistance in *Enterobacteriaceae*: Epidemiology

### 3.1. Historical Overview

Table 1 shows the first appearance of the most important β-lactamases in different countries during the 1963–2006 period, with the relative microorganisms in which these enzymes were isolated.

Since the 1990s, *K. pneumoniae* isolates, producing TEM and SHV ESBLs, have become the predominant causes of nosocomial outbreaks worldwide. To different extents, the TEM-10, TEM-12, and TEM-26 or SHV-2, SHV-5, SHV-7, and SHV-12 enzymes were found in most clinical settings [98]. In 2000, CTX-M enzymes carried by *E. coli* isolates have emerged as important causes of resistance in community onset infections and have spread rapidly to become, in the late 2000s, the most prevalent ESBL in *Enterobacteriaceae* [99]. Among the CTX-M-type enzymes, CTX-M-15 dominates in many countries in Europe [100,101,102,103], Asia [99,104,105], Africa [106], and the USA [13,107,108]. Conversely, CTX-M-14 is a leading resistance mechanism in *E. coli* in Southeast Asia, especially in South Korea and Japan [29]. Together with CTX-M-14, CTX-M-15 became prevalent in China [99,105], whereas CTX-M-2 did in South America [29].

At the end of the 1990s and the beginning of the 2000s, the identification of KPC enzymes led to major epidemics caused by CPEs worldwide, and KPC-2 and KPC-3 are by far the most predominant enzymes in *K. pneumoniae* and other *Enterobacteriaceae* species, including the *E. coli* and *Citrobacter* or *Enterobacter* species [109]. Countries with a high prevalence of KPC-producing *K. pneumoniae* are from Southeast Europe (e.g., Italy, Greece, etc.), South America (Brazil, Colombia), or East Asia (especially China) [109].

The NDM, GES, VIM and IMP enzymes also disseminate globally [13]. Since its first description in 2009 in a Swedish patient hospitalized in India [110], NDM-1 has been reported in travelers who underwent medical procedures in India and Pakistan [111]. In 2017, NDM-1 was found in 134 (3.2%) of 4247 CRE isolates submitted to the Centers for Disease Control and Prevention’s National Healthcare Safety Network for analysis [112]. Since then, NDM-producing *Enterobacteriaceae* were isolated in Balkan states [113], China [114], and Tuscany (Italian region) [115]. VIM-producing *Enterobacteriaceae* are mainly reported, in Europe, as a cause of sporadic hospital outbreaks, with VIM-2 being the most common VIM enzyme worldwide [116,117]. Conversely, in Greece, an epicenter of the VIM-type *Enterobacteriaceae*, VIM-1-producing *K. pneumoniae* and *E. coli* predominate, was considered [118,119]. Since 2015, Hungary, Italy, and Spain showed an inter-regional spread, whereas Africa, Taiwan, Mexico, Saudi Arabia, and the USA showed a sporadic spread of VIM-1 or VIM-2 *Enterobacteriaceae* isolates [120]. Originating from *S. marcescens* isolates in South Pacific and Asia [121], IMP enzymes were found predominantly in *K. pneumoniae*, *E. coli*, and *Enterobacter* isolates [13,122] and were distributed worldwide, despite being endemic only in Japan and Taiwan [119,123].

First identified in Turkey in 2001, OXA-48-type enzymes represent the third globally distributed group of carbapenemases, comprising the canonical OXA-48 and its variants OXA-181 and OXA-232 [124]. Outbreaks of OXA-48-producing *Enterobacteriaceae* mainly occurred in Central or Southern Europe countries, particularly France [125] and Spain [126]. OXA-181 spread in the Indian subcontinent, South Africa, and Singapore, or in patients with an epidemiological link to these areas [127,128]. Not surprisingly, OXA-181 is frequently coproduced together with NDM, reflecting its prevalence in India [129,130].

Apart from non-*Enterobacteriaceae* organisms (e.g., *Stenotrophomonas maltophilia*, *Proteus mirabilis*, or *Proteus vulgaris*), some frequently encountered *Enterobacteriaceae*, such as *K. pneumoniae*, *Klebsiella oxytoca*, and *Salmonella* species, are conspicuous by their lack of chromosomal bla_AmpC_ genes [131]. In *E. coli*, the CMY-2-plasmid-mediated resistance is usually associated with a susceptibility to cefepime. However, cefepime-resistant *E. coli* isolates, producing ESAC β-lactamases, may arise through two to four amino acid deletions in the H-10 helix of the CMY-2 [132]. Although the presence of ESBLs can be masked by high-level AmpC production, the emergence of isolates co-harboring plasmid-encoded AmpC and ESBL genes complicates the situation [133].

### 3.2. Molecular Overview

The horizontal transfer of β-lactamase genes through mobile genetic elements (MGEs), such as transposons, insertion sequences, and integrons, plays an important role in the spread of β-lactam resistance genes within *Enterobacteriaceae*. The MGEs from bacteria present in water and soil environments may accelerate the transfer of ESBL genes to animals and humans [134]. ESBL producers have been detected in urban wastewater, sewage [135], sink pipes [136], livestock [137], and, alarmingly, in retail meat [138]. A recent meta-genome analysis showed that the MGE-mediated transmission of ESBL genes across environmental sources, animals, and humans occurs particularly in developing countries [139,140]. Concerning the acquisition of ESBL-producing bacteria via international travel, the highest rates were seen in Asia, particularly South and Southeast Asia. Thus, Asia is a “key epicenter” of ESBL’s genetic evolution and appears to disseminate major CTX-M types through this way [140]. Likewise, the community transmission of NDM-producing *Enterobacteriaceae* could be through cross-contamination either during food preparation or via body fluids [141], or through animal sources [142,143]. The spread of the OXA-48-producing *Enterobacteriaceae* widely occurs due to healthcare or nosocomial transmission, whereas the OXA-48-like and NDM co-producing *Enterobacteriaceae* is alarming because of the potential for community transmission, particularly in countries with poor hygiene [144].

Although outbreaks are either due to a single ESBL-producing isolate or to a single ESBL plasmid carried by unrelated isolates, differences in the prevalence of various ESBL types may relate to how the different genotypes spread out. In particular, TEM and SHV genes seem to be associated with the dissemination of particular clones, leading to an ‘‘epidemic’’ pattern; otherwise, the mechanism by which CTX-M genes disseminate seems to reflect the simultaneous spread of multiple specific clones, leading to an ‘‘allodemic’’ pattern. A multilocus sequence typing analysis allowed for the identification of five major sequence types (STs) in *E. coli* (ST131, ST405, ST38, ST10, and ST648) and three major STs in *K. pneumoniae* (ST11, ST14, and ST15) [145]. The ST131 is an extraintestinal pathogenic *E. coli* isolate considered a “high risk international clone” and significantly associated with CTX-M-15 [13]. Whole-genome sequencing has revealed three main clades (A, B, and C) within ST131, which mainly differ by their *fim*H alleles encoding for type-1 fimbriae: A (mainly *fim*H41), B (mainly *fim*H22), and C (mainly *fim*H30) [146]. While changes in *fim*H alleles likely influences the colonization capabilities by different clades, the CTX-M-15 gene-dependent segregation of clade C into C1 and C2 drives the dissemination of CTX-M-15 in ST131 [13].

Molecular epidemiology analyses showed that the global KPC-producing *Enterobacteriaceae* spread is mainly due to the clonal expansion of *K. pneumoniae* ST258 over time [147,148,149,150]. ST258, the predominant ST within the clonal complex 258 (CC258), consists of two clades with distinct capsule polysaccharide gene regions [110,151,152]. Interestingly, ST258 clades have been associated with the carriage of specific KPC genes: clade I with KPC-2 and clade II with KPC-3 [110]. Conversely, the horizontal transfer of epidemic broad host-range plasmids carrying the NDM gene explains the spread of NDM-producing *Enterobacteriaceae* [153]. A whole-genome sequencing analysis allowed for the tracking of the variation in the NDM genes harboring plasmids, such as IncA/C, IncF, IncH, IncL/M, IncN and IncX, [61,154,155,156,157] and the fluctuation in the NDM gene flanking regions among nonclonal isolates [13,155,158,159,160]. An in-depth analysis revealed that the IMP genes are located in cassettes within class 1 integrons, often carried by IncL/M and IncA/C plasmids [161,162]. Similarly, VIM-encoding genes are strongly associated with class I integrons and, in turn, are integrated within either chromosomes or plasmids. Therefore, the VIM-1/4 genes in *K. pneumoniae* (ST147 and ST11) are located on N compatibility group plasmids, whereas in *E. coli* they are often associated with IncFI/II plasmids [163]. In contrast to other carbapenemase genes, the dissemination of the OXA-48 gene is associated with a single IncL/M plasmid containing the Tn1999 transposon [13,164,165,166].

## 4. β-Lactamase-Mediated Resistance in *Enterobacteriaceae*: Clinical Importance

Over recent years, the burden of β-lactam resistance among *Enterobacteriaceae* has increased significantly in terms of incidence and, importantly, mortality. For example, in Europe, the number of deaths for infections due to third-generation cephalosporin-resistant *E. coli* and *K. pneumoniae* (of which 88% and 85% were ESBL producers, respectively) increased during the 2007–2015 period [167]. In general, the delayed administration of appropriate targeted therapy (often due to lack of active reporting or delay in the acquisition of isolates’ susceptibility testing data) has been associated with increased mortality rates [168,169,170,171].

It became apparent that ESBL-E infections influence the LOS in either conventional hospital wards or intensive care units. In both settings, the LOS was higher and more costly for patients with bloodstream infections (BSIs) due to ESBL-Es than for patients with BSIs due to non-ESBL-E [172]. In addition, non-invasive infections caused by ESBL-E isolates were also associated with a higher mortality and prolonged LOS [173]. The increased detection of ESBL-E isolates has brought an inevitable large usage of carbapenems as a first-line therapy all over the world. Further, the expenditure for antibiotic therapy is significantly higher for ESBL-E BSIs compared to non-ESBL-E BSIs [172]. To date, the use of β-lactam/β-lactamase inhibitors (BL/BLIs), particularly piperacillin/tazobactam in the setting of ESBL-E infections, remains controversial. In patients with ceftriaxone-resistant *E. coli* or *K. pneumoniae* BSIs, a randomized clinical trial failed to demonstrate the noninferiority of piperacillin-tazobactam for a 30 day mortality compared with meropenem [174]. On the other hand, a recent meta-analysis showed no significant differences in the prognosis for BSIs caused by ESBL-E and treated with carbapenems or BL/BLIs, indicating the usefulness of BL/BLIs for empirical and definitive therapies [175]. A multicenter retrospective study performed in Italy between 2016 and 2019 revealed a high clinical success rate in patients who received ceftolozane-tazobactam in empiric and/or targeted therapy for severe infections [176]. Concerning new drugs for a carbapenem-sparing approach, a recent real-life study assessed the role of ceftazidime-avibactam for the targeted treatment of patients with ESBL-E infections in selected cases, but the small study sample limited the drawing of definitive conclusions [177].

Analogously to ESBL-E, the incidence, mortality, and costs associated with infections due to CPEs, especially *K. pneumoniae*, increased during the 2007–2015 period [167]. The proportion of disability-adjusted life-years (DALYs) due to all the carbapenem-resistant bacteria combined increased from 18% in 2007 to 28% in 2015, whereas the proportion of DALYs due to carbapenem-resistant *K. pneumoniae* and carbapenem-resistant *E. coli* combined doubled from 2007 to 2015 [167]. Unfortunately, CRE infections are associated worldwide with a high risk of clinical failure and a 20–40% mortality rate [178,179,180]. In a recent systematic literature review and meta-analysis, CRE infections were found to be associated with a significantly higher risk of overall mortality compared with carbapenem-susceptible *Enterobacteriaceae* (odds ratio, 3.39; 95% confidence interval, 2.35–4.89). This difference is sharper in BSIs and in infections due to *K. pneumoniae* [181]. Once again, inappropriate early-targeted therapy [182,183] and delays in the receipt of microbiologically-documented, appropriate therapies appear to play a crucial role [181].

The development of new, less toxic combinations based on an old β-lactam molecule with a novel β-lactamase inhibitor active on carbapenemases (ceftazidime-avibactam and meropenem-vaborbactam) has provided a better strategy to face CRE infections. A growing body of evidence shows that ceftazidime-avibactam is superior to polymyxins in the treatment of infections due to KPC isolates in terms of efficacy and safety [179,180]. Another old β-lactam molecule with a novel β-lactamase inhibitor is imipenem-relebactam, which showed a good efficacy and encouraging safety profile for the treatment of imipenem-non susceptible infections, including CRE infections [184]. However, due to the inefficacy of these three new drugs against metallo-β-lactamase-producing CREs, polymyxins remain an intriguing option for high-risk patients with infections due to such isolates [185]. Cefiderocol, a new injectable siderophore cephalosporin with activity against a variety of Ambler class A, B, C and D β-lactamases appears to be very promising against infections caused by CREs, including metallo-β-lactamase producers [186].

Finally, CREs such as carbapenem-resistant *K. pneumoniae* are responsible for significantly increased direct medical costs compared to those for other multidrug-resistant bacteria, suggesting higher economic efforts in terms of the LOS and cost of treatment. The cost impact of CRE colonization/infection on healthcare systems is high and potentially threatening to the stability of the healthcare system. For example, a cost evaluation of a CRE outbreak occurring across a few hospitals in the United Kingdom estimated a cost of approximately 1.1 million euros over 10 months [187]. Thus, CRE infections remain a priority in the matter of prevention and control, and experts recommend use of carbapenems only when no other drug alternative is available [188].

## 5. Conclusions

Infections due to ESBL-Es and CREs are ever increasingly associated with a high incidence and mortality. Thus, counteracting the risk of clinical *Enterobacteriaceae* isolates displaying β-lactamase-mediated resistance and of treatment failures has led to an increased use of combination therapies with more toxic drugs. Unfortunately, the consumption of potentially effective drugs represents an even greater push towards further increases in drug resistance and healthcare system costs. Meanwhile, a better understanding of the molecular mechanisms of β-lactamase-mediated resistance and their updated evolution will allow for the right allocation of resources on drug research and infection control strategies. In parallel, an open access to updated data on the drug resistance rates, at both global and local levels, will support clinical decision-making and antibiotic stewardship programs.

## Figures and Tables

**Table 1:** Overview of most important β-lactamases and relative microorganisms at their first appearance (adapted from reference 10 with permission).

Original (Current) Name	Microorganism	Year (Country) of First Isolation
TEM-1	*Escherichia coli*	1963 (Greece)
SHV-1	*Klebsiella pneumoniae*	1972 (Unknown)
Transferable ESBL (SHV-2)	*Klebsiella pneumoniae*	1983 (Germany)
Serine (class A, group 2f)	*Serratia marcescens*	1982 (England)
Carbapenemase (SME-1)	*Serratia marcescens*	1985 (United States)
FEC-1 (CTX-M-1)	*Escherichia coli*	1986 (Japan)
Plasmid-encoded AmpC (MIR-1)	*Klebsiella pneumoniae*	1988 (United States)
Plasmid-encoded MBL (IMP-1)	*Serratia marcescens*	1988 (Japan)
Inhibitor-resistant TEM (TEM-30)	*Escherichia coli*	1991 (France)
KPC-type (KPC-2)	*Klebsiella pneumoniae*	1996 (United States)
NDM-1	*Klebsiella pneumoniae*	2006 (India)
